# Detection of Recombinant Proteins SOX2 and OCT4 Interacting in HEK293T Cells Using Real-Time Quantitative PCR

**DOI:** 10.3390/life13010107

**Published:** 2022-12-30

**Authors:** Darkhan Kanayev, Diana Abilmazhenova, Ilyas Akhmetollayev, Aliya Sekenova, Vyacheslav Ogay, Arman Kulyyassov

**Affiliations:** Limited Liability Partnership “National Center for Biotechnology” Ministry of Healthcare of the Republic of Kazakhstan, 3/5, Kurgalzhynskoye Road, Astana 010000, Kazakhstan

**Keywords:** biotin ligases, Biotin Acceptor Peptide (BAP), liquid chromatography tandem mass-spectrometry (LC-MS/MS), Proximity Utilizing Biotinylation (PUB), reverse transcription polymerase chain reaction (RT-PCR), transcription factors of pluripotency, western blotting

## Abstract

In vivo biotinylation using wild-type and mutants of biotin ligases is now widely applied for the study of cellular proteomes. The commercial availability of kits for the highly efficient purification of biotinylated proteins and their excellent compatibility with LC-MS/MS protocols are the main reasons for the choice of biotin ligases. Since they are all enzymes, however, just a very low expression in cells is required for experiments. Therefore, it can be difficult to perform the quantifications of these enzymes in various samples. Traditional methods, such as western blotting, are not always fit for the detection of the expression levels. Therefore, real-time qRT-PCR, a technology that is more sensitive, was used in this study to quantify the expression of BirA fusions. Using this method, we detected high expression levels of BirA fusions in models of interactions of pluripotency transcription factors to carry out their relative quantification. We also found the absence of the competing endogenous proteins SOX2 and OCT4, as well as no cross-reactivity between BAP/BirA and the endogenous biotinylation system in HEK293T cells. Thus, these data indicated that the high level of biotinylation is due to the in vivo interaction of BAP-X and BirA-Y (X,Y = SOX2, OCT4) in the cell rather than their random collision, a big difference in the expression level of BirA fusions across samples or endogenous biotinylation.

## 1. Introduction

Protein–protein interactions (PPI) play a key role in many cellular processes, and their detection and quantification are very important for understanding cellular functions [1,2]. Recently, mutant versions of biotin ligases, such as BioID [3], TurboID [4], AirID [5] and BASU [6], have been widely used to identify proteins that are in proximity to the protein of interest (POI) in vivo [7,8]. A common feature of these enzymes is the ability to generate and release biotinoyl-5′-AMP, which is an active form of biotin. This reactive product reacts rapidly with lysine residues of proximal proteins. This makes it possible to efficiently purify these biotinylated proteins using streptavidin beads and identify interacting (or proximal) partners of POI by LC-MS/MS [9].

In contrast, wild-type BirA, a bacterial biotin ligase, retains biotinoyl-5′-AMP and releases it only upon direct contact with specific substrates such as Avitag [10,11] or Biotin Acceptor Peptide (BAP) [12,13]. Thus, it allows for the quantification of PPI, and the use of the AP/BirA pair was first demonstrated by Fernandez et al. [12]. A modified version called the Proximity Utilizing Biotinylation (PUB) method was developed to reduce background biotinylation, and it included an option for the LC-MS/MS detection/quantification of BAP [13]. This method was tested on many model systems, including nuclear proteins such as histones and transcription factors [13,14,15]. The PUB method is not only limited to the testing of PPI. The in vivo creation of a biotin label as a result of the interaction allows for the easy isolation of proteins (or their complexes) from cell lysates in harsh conditions using commercially available streptavidin beads. The benefits of the PUB over just the co-expression of BAP-X and the unconjugated biotin ligase BirA method are more thoroughly discussed in a recent study [16]. For example, modifications of this method, such as PUB-NChip [15], topokaryotyping [17] or its use in labelling at a specific locus in the nucleus [18], can provide valuable information about the structural organization within the cell, which can complement methods using BioID or TurboID.

However, unlike BioID and similar methods, the PUB method requires the expression of two proteins, BAP-X and BirA-Y. Since BirA is an enzyme, the expression of the BirA-Y conjugate in the cell is required at much lower levels than BAP-X. This leads to problems in detecting and quantifying BirA-Y in various samples by western blotting or mass spectrometry. Another problem is that endogenous X and Y proteins may be present in the cell, and their competitive effect on BAP-X and BirA-Y may distort the results of the experiments. In addition to endogenous X and Y, endogenous biotin ligases such as mammalian holocarboxylase synthetase (HCS) and their substrates, which are various carboxylases, are also present in eukaryotic cells [19,20]. This may lead to cross-reactions involving BAP and the bacterial BirA ligase.

The purpose of this work was to help clarify these issues and consisted of the following tasks:

- verify the presence or absence of the expression of the endogenous proteins SOX2 and OCT4;
- test the specificity of the BirA/BAP pair in the presence of the endogenous background biotinylation;
- compare the amount of BirA-Y (SOX2, OCT4) in the experiment (interacting proteins) and in the control (non-interacting proteins) by real-time qRT-PCR.

## 2. Materials and Methods

### 2.1. Materials

Dulbecco’s Modified Eagle’s Medium (DMEM), containing 10% (vol/vol) FBS and 1× penicillin–streptomycin, was prepared by adding 50 mL of FBS (PAN biotech, #P30-3306) and 4.5 mL of penicillin–streptomycin (100×) to 400 mL of DMEM medium. The following were used: Biotin (Sigma-Aldrich, St. Louis, MO, USA, #B4501-1G); TRIzol™ Reagent, (ThermoFisher, Norcross, GA, USA, #15596026); NuPAGE Novex 4–12% Bis-Tris Protein Gels, 12-well (ThermoFisher, #NP0322BOX); cDNA Synthesis kit SuperScript VILO (Invitrogen, #11754-050); cDNA Synthesis kit Reverta-L (#K3-4-100); 1.2 mM phenylmethylsulfonyl fluoride (PMSF) (Applichem, Germany, #APA0999.005); 1 protease inhibitor cocktail PIC (Sigma-Aldrich, St. Louis, MO, USA, #P8340-5ML). A Carl Zeiss Axioobserver A1 inverted research microscope was used for the monitoring of GFP expression and the control of purity of the nuclear fraction during cell disruption. Rabbit polyclonal antibodies to transcriptional factor Sox2, with a molecular weight of 35 kDa, had a high specificity of binding in the immunoblotting assay. Additionally, polyclonal antibodies to Sox2 demonstrated a high nuclear expression in pluripotent stem cells that were determined by fluorescent staining [21]. The HEK293T (Human embryonic kidney) cell lines used for the experiments were from the American Type Culture Collection (ATCC). The pcDNA3.1(+)-BAP-HP1 and pOz-BirA-HP1 [13] vectors were used for the construction of new expression vectors, containing the genes of *SOX2* and *POU5F1* fused with BAP and BirA. The insert sequences were confirmed by sequencing. The vector plasmids pcDNA3-BAP-SOX2 and pOz-humBirA-GFP are available from Addgene (Addgene ID 133281 and 133283, respectively).

### 2.2. Primers Used for Real-Time qRT-PCR

Details can be found in Supplementary Table S1.

### 2.3. Cell Culture

For cell preparation, the tubes containing HEK293T cells were thawed at 37 °C. Then, the cells were transferred to a 15 mL tube filled with PBS and centrifuged at 300× *g* for 5 min. The supernatant was removed, and 3 mL of the cells was resuspended in the DMEM medium. The cell suspension was transferred to a T75 flask, and additional DMEM was added up to 8 mL and placed in an incubator at 37 °C (CO_2_-4.8%). The cells were cultured in T75 flasks, changing the medium regularly until the cell confluence reached 90%.

To seed the cells in dishes, the DMEM medium was removed from the T75 flask and washed with 10 mL PBS. The cells were then trypsinized by adding 4.5 mL PBS and 0.5 mL trypsin and incubated at 37 °C for 3 min. The trypsinized cells were centrifuged at 300× *g* for 5 min. Then, the supernatant was removed, and 5 mL of the DMEM medium was added and resuspended. After counting, the cells were transferred to 10 cm dishes. Then, 1.5–2.0 × 10^6^ cells were added to each dish (2 dishes for each cell line) containing 5 mL of the DMEM medium.

### 2.4. Transient Transfection by LIPOFECTAMINE 2000 (General Procedure, X,Y = SOX2, OCT4)

Media in the 10 cm dishes containing cells were replaced with fresh DMEM at least one hour before transfection. For transfection, 1250 µL Opti-MEM was premixed with 34.48 µL (3 µg) pOz-BirA-Y plasmid. Then, the diluted pOz-BirA-Y plasmid was divided into two 625 µL tubes. A total of 11.4 µL (3 µg) of the CMV-BAP-GFP plasmid was added to the first tube, and 6.2 µL (3 µg) of the CMV-BAP-X plasmid was added to the second tube. In two separate tubes, 650 µL of Opti-MEM was mixed with 28.6 µL of Lipofectamine 2000 reagent and incubated for 5 min at room temperature. The plasmid mixtures were added to the corresponding tubes with Lipofectamine 2000 reagent and were allowed to stand at room temperature for 20 min. Then, 625–650 µL of the DNA–lipid complex was added dropwise to each dish containing cells with 4.3 mL Opti-MEM medium. The dishes were incubated at 37 °C in a CO_2_ incubator, and after 4–6 h, the Opti-MEM medium was replaced with the DMEM medium.

### 2.5. Biotin Labeling and Cell Harvesting

For biotin labeling, a medium containing 30 mL DMEM, 1500 µL HEPES and 150 µL biotin was prepared. Then, the medium was replaced with the biotin-containing DMEM medium 3–6 h before the cell harvest. The final concentration of biotin was 5 μg/mL. Before the cell collection, the DMEM medium was removed and replaced with 1× PBS buffer. The cells were resuspended and transferred to Eppendorf tubes. Before centrifugation at 4000 rpm for 5 min, 1/5 of the cell suspension was aliquoted for western blotting, and the rest were for RNA isolation with Trizol. The cells for the western blotting were stored at −20 °C, and the cells for the qRT-PCR were flash-frozen in liquid nitrogen and stored at −70 °C.

### 2.6. Western Blotting (General Procedure, X,Y = SOX2, OCT4)

The western blotting was performed for three cell samples: Sample 0, 0′ control, Sample 1, 1′ (BAP-GFP + BirA-Y) and Sample 2, 2′ (BAP-X + BirA-Y).

Each sample cell was sonicated with 100 µL of CSK buffer (PMSF). They were prepared for electrophoresis by adding 4X LDS buffer (50 mM DTT) and denaturing them at 95 °C for 5 min. The gel electrophoresis was conducted using two gels in a MOPS buffer. The proteins were then transferred on nitrocellulose membranes (total: six) using a transfer. The membranes were blocked using a solution containing PBS with 0.2% Tween and 5% milk overnight.

After thorough washing, two membranes were incubated in 10 mL of PBS with 0.2% Tween, 0.125% milk and 2 μL streptavidin-HRP or anti-His-HRP for 1 h in the dark. The other four membranes were first incubated in 10 mL of PBS with 0.05% Tween and 0.125% milk solution with primary antibodies (2 μL of SOX2, OCT4, NANOG, 10 μL of C-MYC) for 2 h, washed and incubated again with the secondary antibody (Anti-Rabbit) for 1 h (in the dark).

After washing, the membranes were treated with a mixture of 1000 μL Luminol/enhancer solution (Solution A), 1000 μL Stable peroxide (Solution B) buffers and 200 µL 3% hydrogen peroxide solution and developed on a film with a threefold increase in the exposure time (10, 30, 90 and 300 s).

### 2.7. RNA Isolation with TRIZOL Reagent from HEK293T

The RNA isolation from cells was performed according to the standard protocol [22].

### 2.8. Reverse Transcription (General Procedure, X,Y = SOX2, OCT4)

To obtain cDNA from RNA with the Reverta-L kit, a reaction mixture for three reactions—Sample 0, 0′ control, Sample 1, 1′ (BAP-GFP + BirA-Y) and Sample 2. 2′ (BAP-X + BirA-Y)—was prepared.

To prepare the reaction mixture, 31.4 µL of RT-mix and 1.25 µL of RT-G-mix1 were added to the tube. They were thoroughly mixed on a vortex and spun on a minicentrifuge. Then, 1.5 μL of revertase (MMlv) was added to the resulting solution, pipetted five times, vortexed and spun. The reaction mixture was divided into three microtubes, each containing 10 µL of the mixture. Then, 10 µL of the RNA probes was added to each microtube containing the reaction mixture and pipetted. The test tubes were placed in an amplifier at 37 °C for 30 min.

### 2.9. Real-Time qRT-PCR

Real-time PCR was performed for three cDNA samples (samples 0, 1, 2 or 0′, 1′, 2′) with the housekeeping gene (Actin) and two primer mixes (OCT4, SOX2). Three replicate experiments were performed for each primer mix (OCT4, SOX2).

The reaction mix for the housekeeping gene was prepared for three reactions, while the reaction mixes for OCT4 and SOX2 were prepared for nine reactions each (triplet). Each microtube contained the master mix, the final concentrations of SOX2 (or OCT4) Forward Primer, SOX2 (or OCT4) Reverse Primer and SOX2 (or OCT4) TaqMan probes, 5 U of Taq polymerase and 100 ng of cDNA samples (0, 1, 2 or 0′, 1′, 2′) in a total volume of 20 µL.

The samples were placed in the thermocycler with the following conditions: initialization at 94 °C for 3 min (by hot-start PCR), denaturation at 94 °C for 10 s, 40 cycles of annealing and elongation at 58 °C for 40 s for OCT4. In the case of SOX2, the elongation step was conducted at 64 °C. The SOX2 annealing temperature (64 °C) was selected depending on the primer design. The primers were designed using the FastPCR program [23,24], which is freely available at http://primerdigital.com, accessed on 30 September 2022.

## 3. Results and Discussion

The main requirement for correct results in the PUB method is that the concentration of the expressed enzyme in the cells must be significantly lower than the concentration of the target (BirA-Y << BAP-X), e.g., in reactions with a pseudo-first-rate order [11,25]. This is achieved by using plasmids with weak and strong promoters during transfection. The difference in recombinant protein expression was visually demonstrated in experiments with the transfection of the enhanced GFP reporter protein, expressed under strong CMV and weak MoMuLV promoters (Supplementary Figure S1).

Previously, we performed experiments to quantify the protein–protein interactions of SOX2 and OCT4 in vivo, using western blotting and mass spectrometry in the MRM mode [14]. However, we did not test whether the endogenous proteins SOX2 and OCT4 were present in the cells, which could affect the interaction of BAP-X and BirA-Y (X, Y = SOX2, OCT4) in sample 2 due to the competition effect.

Another important issue is the biotinylation specificity for the pair BirA/BAP. In the PUB method, we use vectors containing the humanized bacterial BirA biotin ligase sequence, where the nucleotide sequence is codon-optimized for expression in mammalian cells [26]. Therefore, it was necessary to test whether BirA can biotinylate endogenous proteins in a eukaryotic cell. Conversely, can the BAP target be a substrate for the endogenous biotin ligase of HEK293T cells?

Another feature in these experiments was the nonexistent or very weak biotinylation in the control BAP-GFP + BirA-OCT4 (sample 1), which increased with the longer labeling with biotin (up to 9 h). A possible reason for this could be the much higher level of BirA-OCT4 expression in sample 2 compared to that in sample 1. The expression of this protein could be assessed using western blotting since there is an 8×His-tag between BirA and OCT4. Unfortunately, the expression of BirA-OCT4 was not sufficient to be detected by western blotting. Therefore, we used the real-time qRT-PCR method for the comparative quantitative analysis of protein expression.

To solve these problems, additional experiments were performed in this work, such as:

- Testing the level of expression of endogenous SOX2 and OCT4 proteins in cells;
- Introducing additional controls showing the absence of BAP biotinylation due to endogenous biotin ligases from HEK293T cells;
- Quantitatively comparing the expression level of BirA fusions in the control and experiment using real-time qRT-PCR.

### 3.1. Testing the Level of Expression of Endogenous SOX2 and OCT4 Proteins in Cells

**Figure 1:**
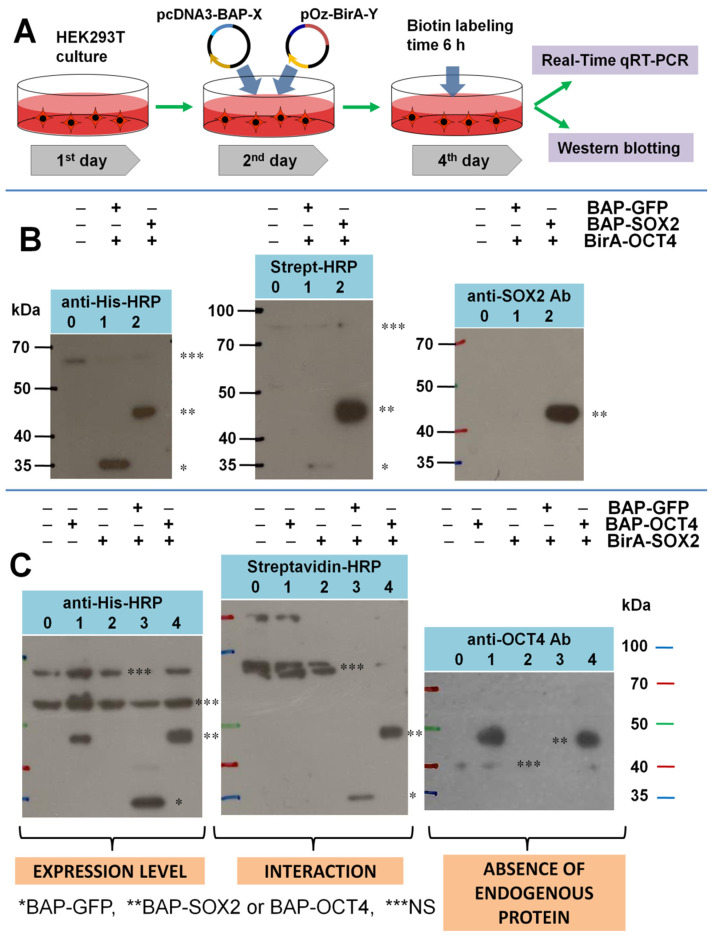
Validation of the interaction of BAP-X and BirA-Y. (**A**) Experimental workflow for the detection of protein–protein interactions between SOX2 and OCT4. (**B**) Western blots of the experiment where X = SOX2 and Y = OCT4. The positions of BAP-fusions and nonspecific signals (NS) are indicated by asterisks. The biotin labeling time was 3 h. The BAP fragment has a His-tag, and the first western blot shows comparable total amounts of BAP-GFP and BAP-SOX2. The Streptavidin blot shows the number of biotin-labeled BAP-fusions which are the result of protein–protein interactions (or proximity) of BAP-SOX2 and BirA-OCT4 in lane 2. A very strong signal was observed in lane 2, and a very weak signal was observed in lane 1, which may be the result of a random collision of BAP-GFP and BirA-OCT4. Treatment with an anti-SOX2 antibody on the third blot indicates recombinant BAP-SOX2 and the absence of a detectable amount of endogenous SOX2. BAP adds a mass shift of 3 kDa, and only a single band was observed on the anti-SOX2 blot in lane 2. (**C**) Western blots of the reciprocal experiment where X = OCT4 and Y = SOX2. The biotin labeling time was 6 h. A comparable expression of BAP-GFP and BAP-OCT4 was detected on anti-His blot lanes 3 and 4. The left blots show the expression, the middle blots show the interaction and the right blots show the absence of the endogenous proteins SOX2 or OCT4. Note that BirA-X (which is actually BirA-8×His-tag-X) fusions were not observed on anti-His blots.

The standard workflow of the PUB experiment is shown in Figure 1A. Using the lipofectamine, HEK293T cells were transfected by two plasmids: pcDNA3-BAP-SOX2 and pOz-BirA-OCT4 (Figure 1B and Figures S2–S4). In the reciprocal experiment, we used plasmids with reverse combinations of corresponding ORF: pcDNA3-BAP-OCT4 and pOz-BirA-SOX2 (Figure 1C and Figure S5). Cells were labeled by adding biotin to the DMEM medium before harvesting (for example, 6 h of biotin labeling time). The cells were then divided into two parts, one for qRT-PCR analysis and the other for western blotting.

We determined the endogenous protein expression by western blotting with antibodies against the pluripotency transcription factors SOX2, OCT4, NANOG and C-MYC. As shown in Figure 1B, only the expression of the recombinant BAP-SOX2 protein was detected (right blot). The characteristic double bands (BAP-SOX2 + endogenous SOX2) were not observed, which were previously detected in another experiment with the expression of BAP-PCNA and endogenous PCNA upon treatment with antibodies against PCNA [15].

In the reciprocal experiment (Figure 1C), we also did not observe the expression of the endogenous OCT4 protein. Only bands corresponding to BAP-OCT4 were observed in lane 1 (where only BAP-OCT4 was expressed) and lane 4 (where BAP-OCT4 was expressed together with BirA-SOX2).

Bands corresponding to other endogenous transcription factors (NANOG, c-Myc and the recombinant proteins BirA-SOX2 and BirA-OCT4) were not observed (Supplementary Figure S4, uncropped blot C).

### 3.2. Testing the Specificity of Biotinylation for the BAP/BirA Pair in HEK293T Cells

It is known that endogenous biotin ligases biotin-protein ligase (BPL), also known as holocarboxylase synthetase (HCS), are expressed in mammalian cells [19,20], as well as their substrates, such as acetyl-CoA-carboxylase [27], pyruvate carboxylase [28], propionyl-CoA carboxylase [19] and methylcrotonyl-CoA carboxylase [29], which could affect the specificity of BirA/BAP biotinylation. Therefore, we used additional controls in which BAP-X and BirA-Y were expressed in separate dishes. The western blot for anti-His showed a comparable amount of BAP-SOX2 in samples 2 and 4, but no biotinylation was observed in 2, where only BAP-SOX2 was expressed (Supplementary Figure S2). The similarity of the biotinylation patterns in samples 0 and 1 also indicated that BirA-OCT4 does not noticeably biotinylate endogenous proteins. Similarly, in the reciprocal experiment, we did not observe the biotinylation of BAP-OCT4 in the absence of BirA-SOX2 (lane 1, on the streptavidin blot in Figure 1C). These results are consistent with previously published data [30] that showed a lower activity of mammalian HCS towards bacterial BCCP87 compared to its native substrate p-67. To reduce the background BAP biotinylation, the peptide used in the PUB method was intentionally designed to have lower substrate specificity for BirA than the Avitag peptide [13,16]. Therefore, the biotinylation of BAP by the endogenous biotin-protein ligase HCS was not observed in the absence of bacterial BirA.

### 3.3. Quantitative Comparison of the Expression Level of BirA-Y (Y = SOX2, OCT4) Fusions Using Real-Time qRT-PCR

**Figure 2:**
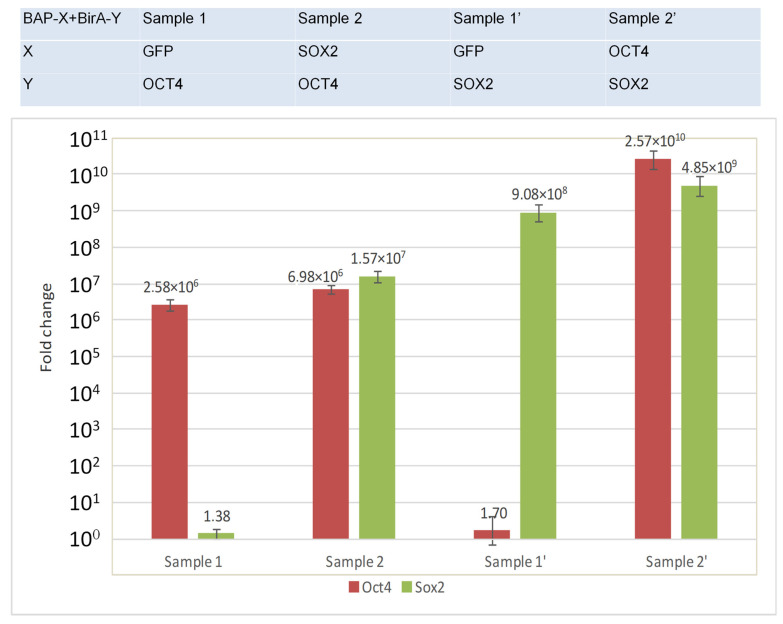
Real-time qRT-PCR amplification of OCT4 and SOX2 in the PUB experiments. Error bars represent the standard deviation.

Despite a number of advantages, western blotting also has some limitations. In our case, this is due to the impossibility of detecting BirA fusions, which is related to the limited sensitivity of this method. For example, the most sensitive variant, using enhanced chemiluminescence (ECL), can detect up to 1–3 pg of the antigen [31]. In contrast, the real-time qRT-PCR method has a much higher sensitivity. For example, it was recently demonstrated that this method can be used to detect the SARS-CoV-2 virus with a lower limit of detection of up to eight copies per ml [32]. Indeed, we were able to detect and comparatively quantify BirA fusions using real-time qRT-PCR (Figure 2).

The expression of the OCT4 gene was detected in sample 1 (BAP-GFP + BirA-OCT4) and sample 2 (BAP-SOX2 + BirA-OCT4) but not in sample 0 (control without plasmids). This means that the signal was due to the presence of BirA-OCT4 but not endogenous OCT4. In addition, the levels of expression of OCT4 in both samples were comparable (Figure 2). The expression of OCT4 in sample 1 was detected at cycle 19, while for sample 2, it was seen at cycle 18 (approximately 2–2.5 times difference).

The expression of the SOX2 gene was detected only in sample 2 (BAP-SOX2 + BirA-OCT4). In sample 2, where both genes were found, the expression level of Sox2 is higher than that of OCT4. Real-time PCR showed the expression of target genes in samples with transfected plasmids, while no target gene was shown in control sample 0.

Similarly, in the reciprocal experiment (Figure 2, right bar chart diagram), we observed the presence of BirA-SOX2 in samples 1′ and 2′ and the absence of SOX2 expression in the control.

Thus, the RT PCR data showed that the expression level of BirA-Y was comparable in samples 1 and 2 (1′ and 2′). The absence of the SOX2 and OCT4 signal in the control samples 0 and 0′ indicated that the amplification in samples 1 and 2 was due to the presence of BirA-Y alone.

## 4. Conclusions

This study has shown the feasibility of using the ‘TaqMan’ fluorescence probe-based real-time qRT-PCR assay to quantitatively compare the expression of BirA-Y (Y = SOX2, OCT4) biotin ligase fusions in different samples. This is important because the weak biotinylation may be a result of low BirA-Y expression in the control sample. Western blotting with streptavidin conjugates provides information on the degree of biotinylation but is only an indirect confirmation of the result of BirA-Y enzymatic activity. A real–time qRT-PCR assay is a more sensitive method because it relies on cDNA amplification. Thus, by using primers for SOX2 and OCT4, we were able to simultaneously determine the expression of both of the recombinant BirA-Y proteins and the absence of the corresponding endogenous proteins, which was also confirmed by western blotting analysis.

## Data Availability

The vector plasmids pcDNA3-BAP1070-SOX2 and pOz-BirA-GFP were deposited in the Addgene repository (ID 133281 and 133283). All other plasmid constructs used during the current study are available from the corresponding author on request: Synthetic construct enhanced green fluorescent protein (eGFP) gene, partial cds (GenBank: MN443913.1), synthetic construct BAP1070-SOX2 (GenBank: MW281044.1), synthetic construct BAP1108-SOX2 (GenBank: MW281045.1).

## Supplementary Materials

The following supporting information can be downloaded at: https://www.mdpi.com/article/10.3390/life13010107/s1, Figure S1. Comparison of the expression of eGFP under strong CMV (A) and weak MoMuLV (B) promoters. Figure S2. Experiment with additional controls for testing the specificity of biotinylation. Figure S3. Uncropped blot from Figure S2. Figure S4. Uncropped blot from Figure 1B of the manuscript. Figure S5. Uncropped blot from Figure 1C of the manuscript (Reciprocal experiment). Figure S6. Bar chart diagram and overlay of amplification plots for samples 0–2. Figure S7. Bar chart diagram and overlay of amplification plots for samples 0′–2′. Table S1. List of Gene Primers (the Forward and Reverse Sequences and the TaqMan probes), Annealing Conditions and Region of Amplification for the RT PCR. Table S2. Calculation of OCT4 expression in samples 0–2. Table S3. Calculation of SOX2 expression in samples 0–2. Table S4. Calculation of OCT4 expression in samples 0′–2′. Table S5. Calculation of SOX2 expression in samples 0′–2′.
