# Supplementary material for: Detection of Recombinant Proteins SOX2 and OCT4 Interacting in HEK293T Cells Using Real-Time Quantitative PCR
Source: Life (Basel). 2022 Dec 30;13(1):107. doi: 10.3390/life13010107 (PMC9862934; doi:10.3390/life13010107)
Supplement: Supplementary file 1 [file life-13-00107-s001.zip › life-2029193-supplementary.pdf]

Supplementary data

Detection of Recombinant Proteins SOX2 and OCT4 Interacting in HEK293T Cells Using Real-Time Quantitative PCR

Darkhan Kanayev, Diana Abilmazhenova, Ilyas Akhmetollayev, Alia Sekenova, Vyacheslav Ogay and Arman Kulyyassov*

[1] Limited liability partnership "National Center for Biotechnology" Ministry of Healthcare of the Republic of Kazakhstan, 3/5, Kurgalzhynskoye Road , Astana, 010000, Kazakhstan
* Correspondence: kulyyasov@biocenter.kz; Tel.: +7-7172-707534

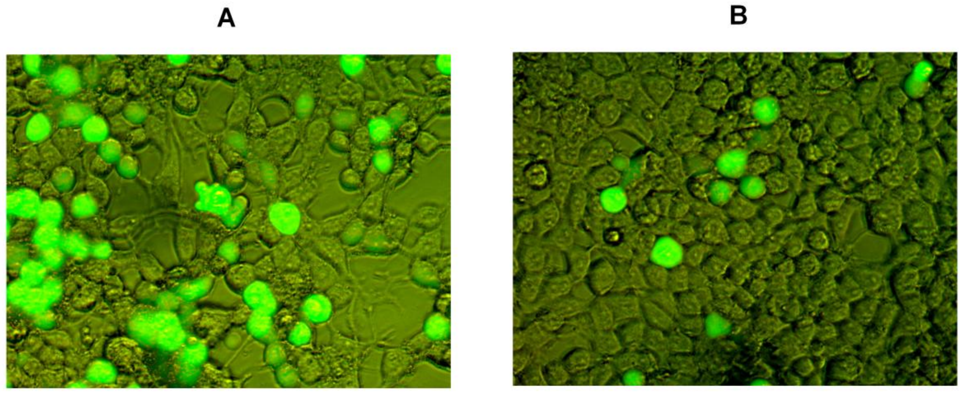

Figure S1. Comparison of expression of eGFP under strong CMV (A) and weak MoMuLV (B) promoters. HEK293T cells were transfected by corresponding plasmids (CMV-BirA-GFP or MoMuLV-BirA-GFP) using Lipofectamine.

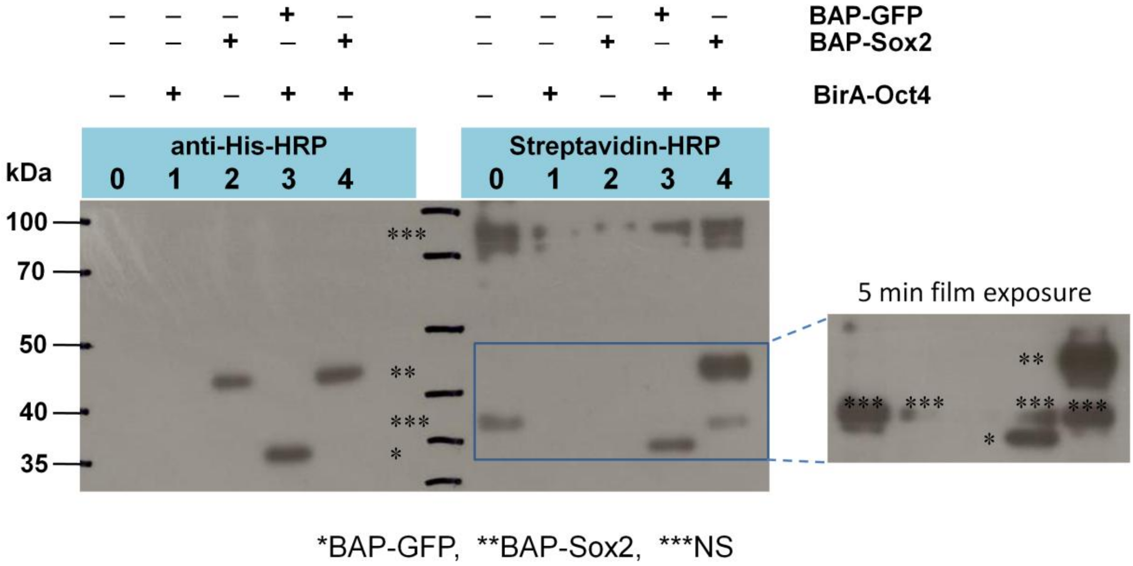

Figure S2. Experiment with additional controls for testing specificity of biotinylation. Sample 0 – no plasmid were added to the cells. BirA-OCT4 and BAP-SOX2 were expressed separately in samples 1 and 2 correspondingly. Biotin labeling time was 9h. The positions of the BAP-fusions and nonspecific signals (NS) are indicated as asterisks. No biotinylation bands except nonspecific bindings were observed in lines 0-2. Biotinylation level of BAP-SOX2 in sample 4 is much higher than BAP-GFP in sample 3. No biotinylation was observed in case of separate BAP-SOX2 or BirA-OCT4 expressions (lanes 1 and 2 on streptavidin blot).

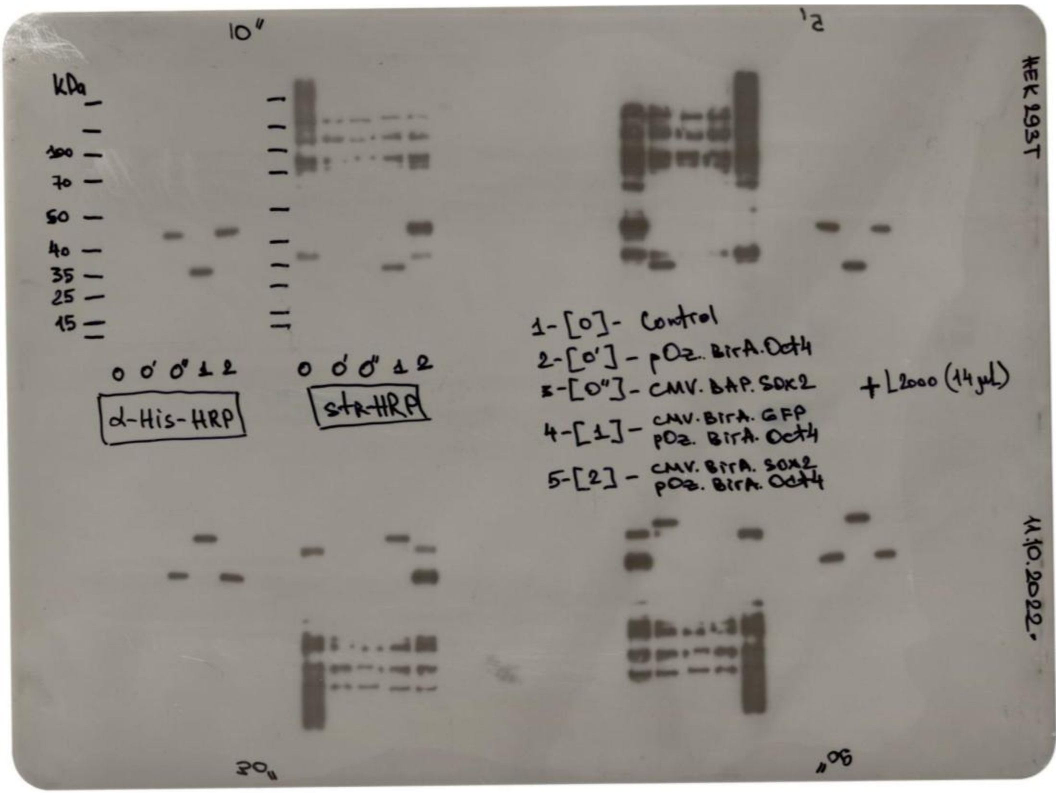

Figure S3. Uncropped blot from Figure S2**.** Testing the specificity of biotinylation for BAP/BirA pair in HEK293T cells. Time of film exposure: 10 sec, 30, sec, 90 sec and 5 min.

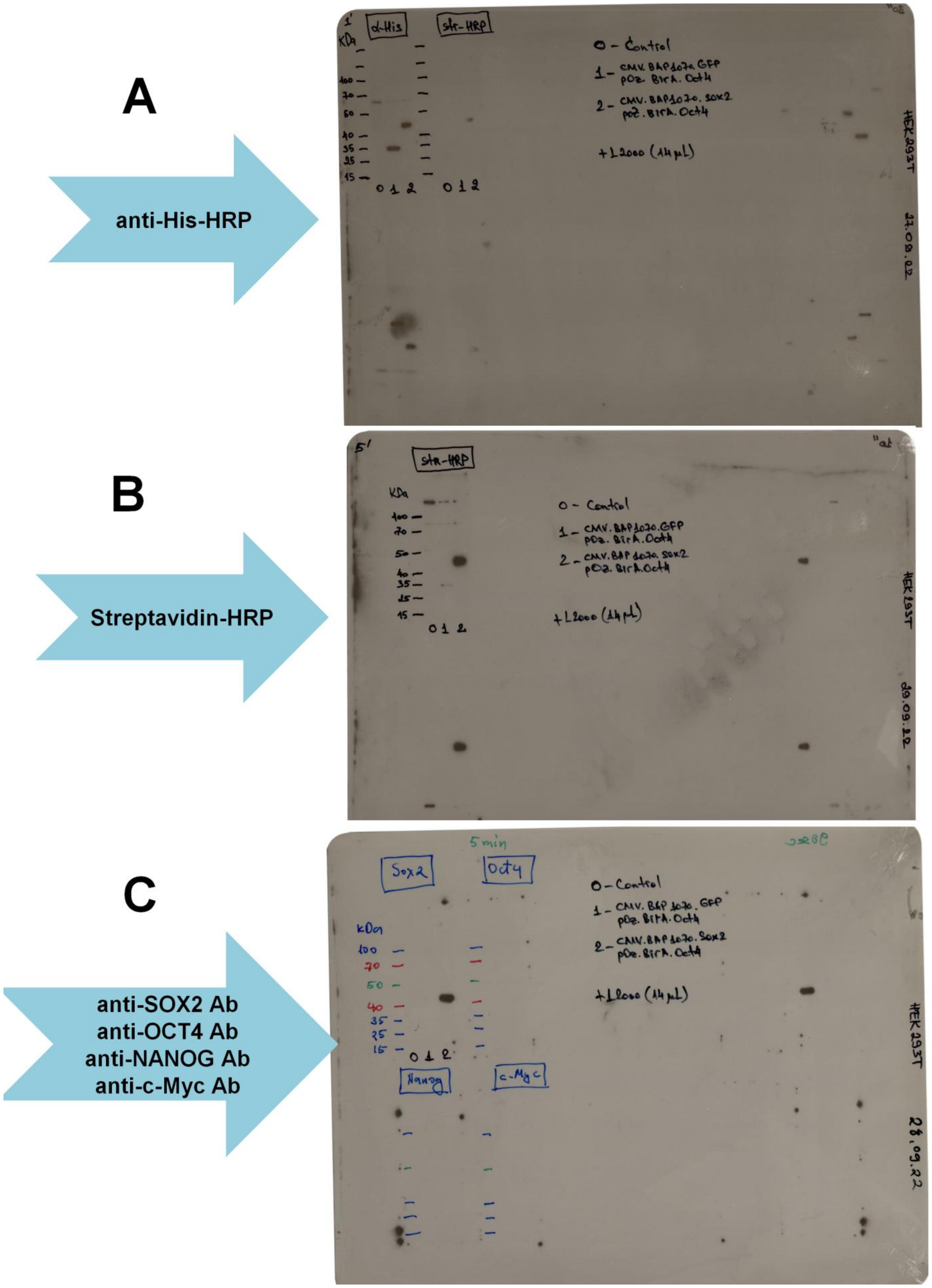

Figure S4. Uncropped blot from Figure 1B of manuscript. Sample 0 – nontransfected cells, sample 1 - BAP-GFP+BirA-OCT4, sample 2 - BAP-SOX2+BirA-OCT4. (A) anti-His antibody. (B) Streptavidin-HRP conjugate. (C) Checking the level of expression of endogenous SOX2, OCT4, NANOG and c-Myc proteins in cells.

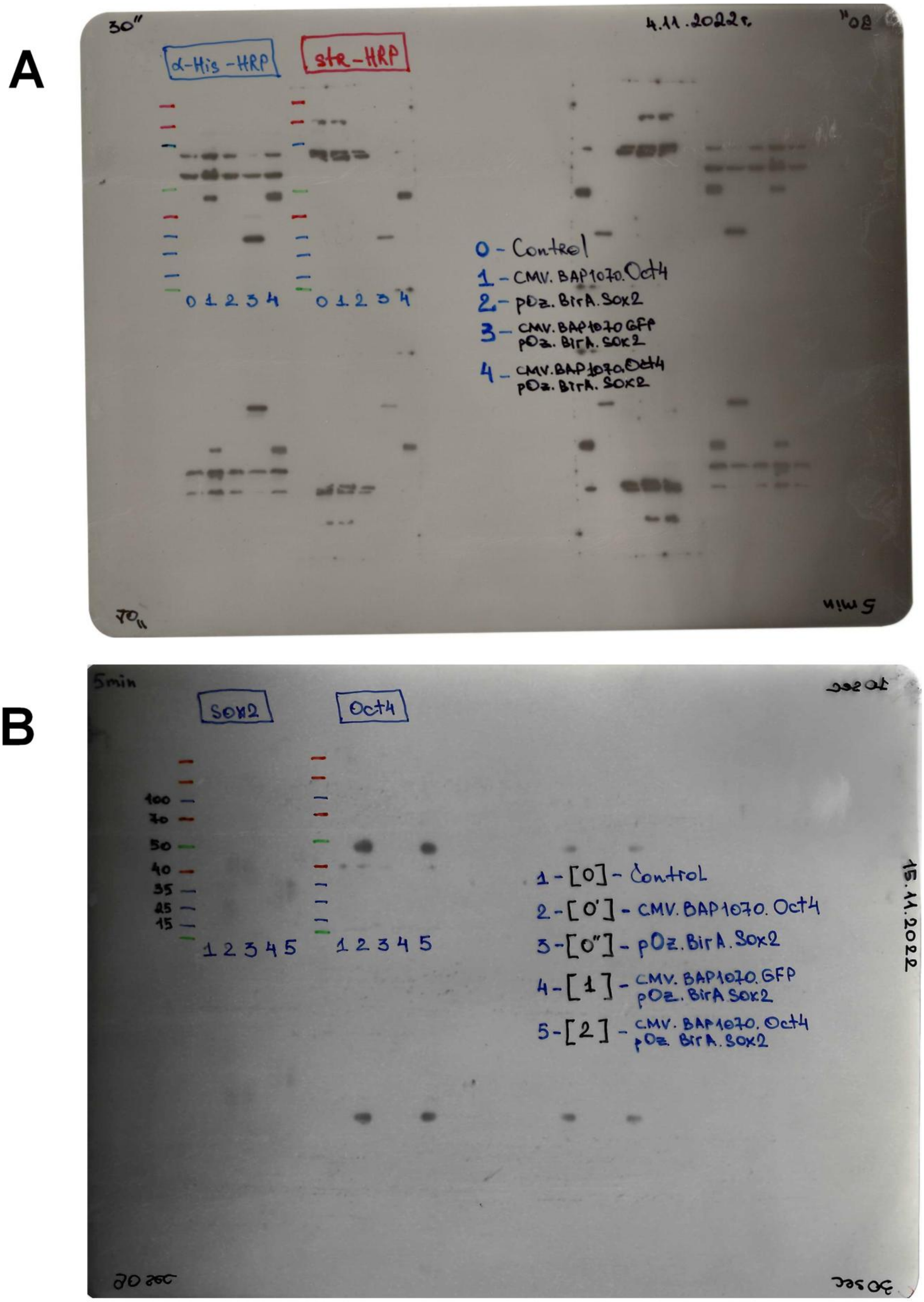

Figure S5. Uncropped blot from Figure 1C of manuscript (Reciprocal experiment). Sample 0 – nontransfected cells, sample 1 - BAP-OCT4 alone, sample 2 - BirA-SOX2 alone, sample 3 - BAP-GFP+BirA-SOX2, sample 4 – BAP-OCT4 +BirA-SOX2. (A) anti-His antibody and Streptavidin-HRP conjugate. (B) Checking the level of expression of endogenous SOX2 and OCT4 proteins in cells.

Real time quantitative RT PCR

Table S1. List of Gene Primers (the Forward and Reverse Sequences and TaqMan probes), Annealing Conditions, and Region of Amplification for the RT PCR.

№	Gene	Sequence 5′-3′	Annealing temperature °C	Primer concentration μM	NCBI accession ID
1	ACTB	TCACCATTGGCAATGAG<br>CCACGTCACACTTCATG<br>ROX-ACTCTTCCAGCCTTCCTTCC-BHQ2	58	0.5	NM_001101
2	SOX2	CTCCGGGACATGATCAGC<br>CTGGGACATGTGAAGTCTGC<br>FAM-TTCCGGCACCTCGGCG-BHQ1	64	0.5	NM_003106
3	OCT4	GGGTTCTATTTGGGAAGG<br>TCCTGAAGATTTTCATTGTTG<br>FAM-ACGACCATCTGCCGCT-BHQ1	58	0.5	NM_001173531

a. The ΔC_T value is determined by subtracting the average OCT4 C_T value from the average Housekeeping (Actin) C_T value.

$$\Delta C_T = C_{T,target} - C_{T,housekeeping}$$

The standard deviation of the difference is calculated from the standard deviations of the target and housekeeping values. $s = \sqrt{{s_{target}}^2 + {s_{houskeeping}}^2}$

b. The calculation of $\Delta\Delta C_T$ involves subtraction by the ΔC_T calibrator value (control). $\Delta\Delta C_T = \Delta C_{T,sample} - \Delta C_{T,control}$ This is subtraction of an arbitrary constant, so the standard deviation of $\Delta\Delta C_T$ is the same as the standard deviation of the ΔC_T value.

c. The range given for the expression of target gene relative to Sample 0 is determined by evaluating the expression: 2 $\Delta\Delta C_T$ with $\Delta\Delta C_T$ + s and $\Delta\Delta C_T$ – s, where s = the standard deviation of the $\Delta\Delta C_T$ value.

Table S2. Calculation of OCT4 expression in samples 0-2.

Sample	Housekeeping (Actin) Average C_t	OCT4 Average C_T	ΔC_T	$\Delta\Delta C_T$	OCT4 Rel. to Sample 0
Sample 0	23,75 ± 0,04	40,00 ± 0,00	16,25 ± 0,04	0,00 ± 0,04	1,00 (0,97 – 1,03)
Sample 1	24,22 ± 0,47	19,17 ± 0,14	-5,05 ± 0,49	-21,30 ± 0,49	$2,58\times10^{6}$ ($1,84\times10^{6}$ – $3,63\times10^{6}$)
Sample 2	24,79 ± 0,36	18,31 ± 0,22	-6,48 ± 0,42	-22,73 ± 0,42	$6,98\times10^{6}$ ($5,23\times10^{6}$ – $9,31\times10^{6}$)

Table S3. Calculation of SOX2 expression in samples 0-2.

Sample	Housekeeping (Actin) Average C_t	SOX2 Average C_T	ΔC_T	$\Delta\Delta C_T$	SOX2 Rel. to Sample 0
Sample 0	23,75 ± 0,04	40,00 ± 0,00	16,25 ± 0,04	0,00 ± 0,04	1,00 (0,97 – 1,03)
Sample 1	24,22 ± 0,47	40,00 ± 0,00	15,78 ± 0,47	-0,47 ± 0,47	1,38 (1,00 – 1,92)
Sample 2	24,79 ± 0,36	17,14 ± 0,39	-7,65 ± 0,53	-23,91 ± 0,53	$1,57\times10^{7}$ ($1,09\times10^{7}$ – $2,27\times10^{7}$)

A

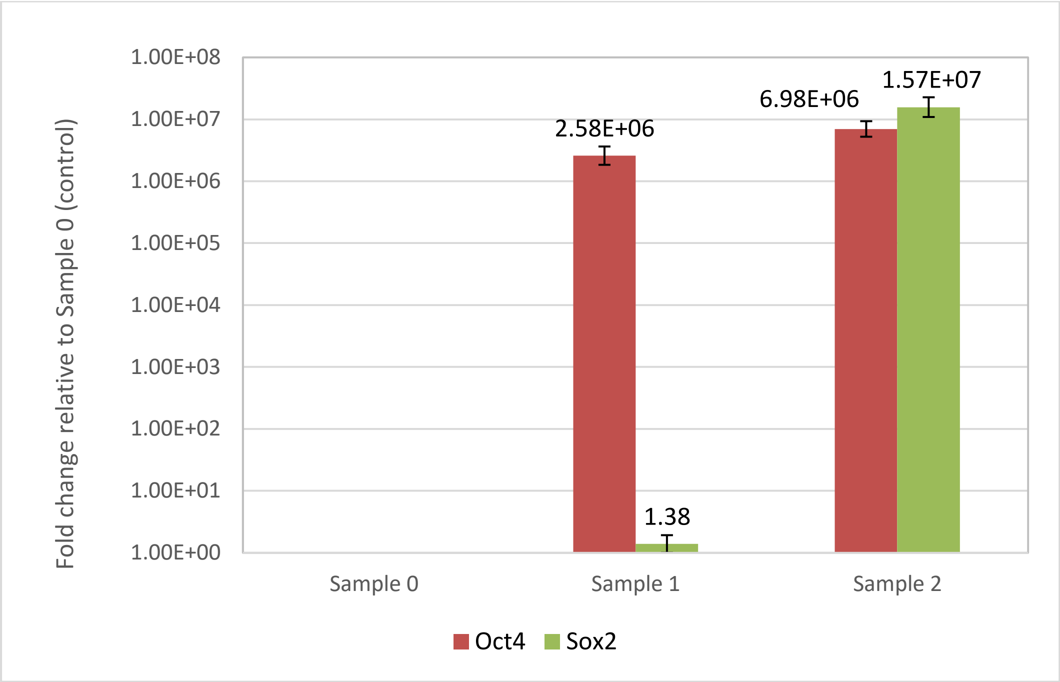

B

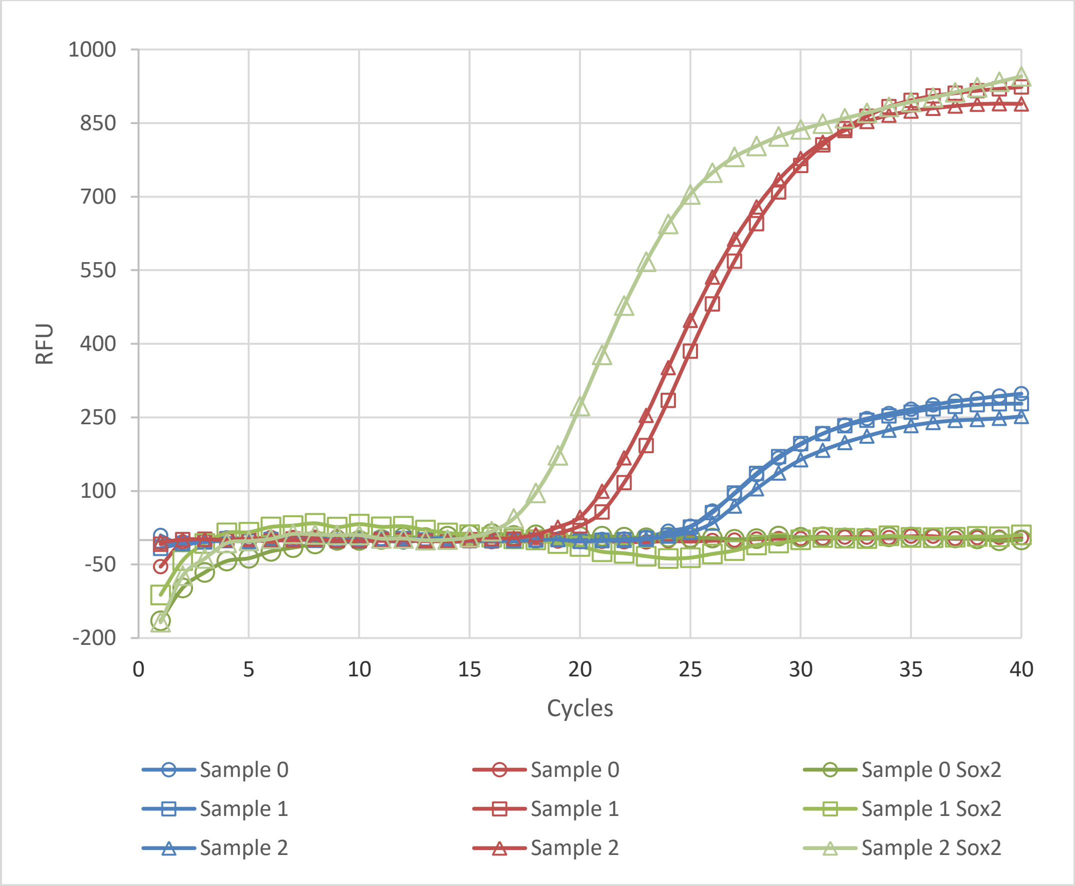

Figure S6. Bar chart diagram and overlay of amplification plots for samples 0-2. Sample 0 (control without plasmids), Sample 1 (BAP-GFP + BirA-OCT4), Sample 2 (BAP-SOX2 + BirA-OCT4): (A) Relative normalized expression of target genes; (B) real-time PCR amplification curves

Table S4. **Calculation of OCT4 expression in samples 0′-2′.**

Sample	Housekeeping (Actin) Average C_t	OCT4 Average C_T	ΔC_T	$\Delta\Delta C_T$	OCT4 Rel. to Sample 0′
Sample 0′	20,28 ± 0,33	40,00 ± 0,00	29,72 ± 0,33	0,00 ± 0,33	1,00 (0,79 – 1,26)
Sample 1′	21,05 ± 1,25	40,00 ± 0,00	28,95 ± 1,25	-0,77 ± 1,25	1,70 (0,72 – 4,03)
Sample 2′	20,75 ± 0,87	15,88 ± 0,17	-4,87 ± 0,88	-34,58 ± 0,88	$2{,}57\times10^{10}$ ($1{,}39\times10^{10}$ – $4{,}74\times10^{10}$)

Table S5. **Calculation of SOX2 expression in samples 0′-2′.**

Sample	Housekeeping (Actin) Average C_t	SOX2 Average C_T	ΔC_T	$\Delta\Delta C_T$	SOX2 Rel. to Sample 0
Sample 0′	20,28 ± 0,33	40,00 ± 0,00	29,72 ± 0,33	0,00 ± 0,33	1,00 (0,79 – 1,26)
Sample 1′	21,05 ± 1,25	20,51 ± 0,53	-0,04 ± 0,75	-29,76 ± 0,75	$9{,}08\times10^{8}$ ($5{,}38\times10^{8}$ – $1{,}53\times10^{9}$)
Sample 2′	20,75 ± 0,87	18,29 ± 0,24	-2,46 ± 0,90	-32,18 ± 0,90	$4{,}85\times10^{9}$ ($2{,}60\times10^{9}$ – $9{,}04\times10^{9}$)

A

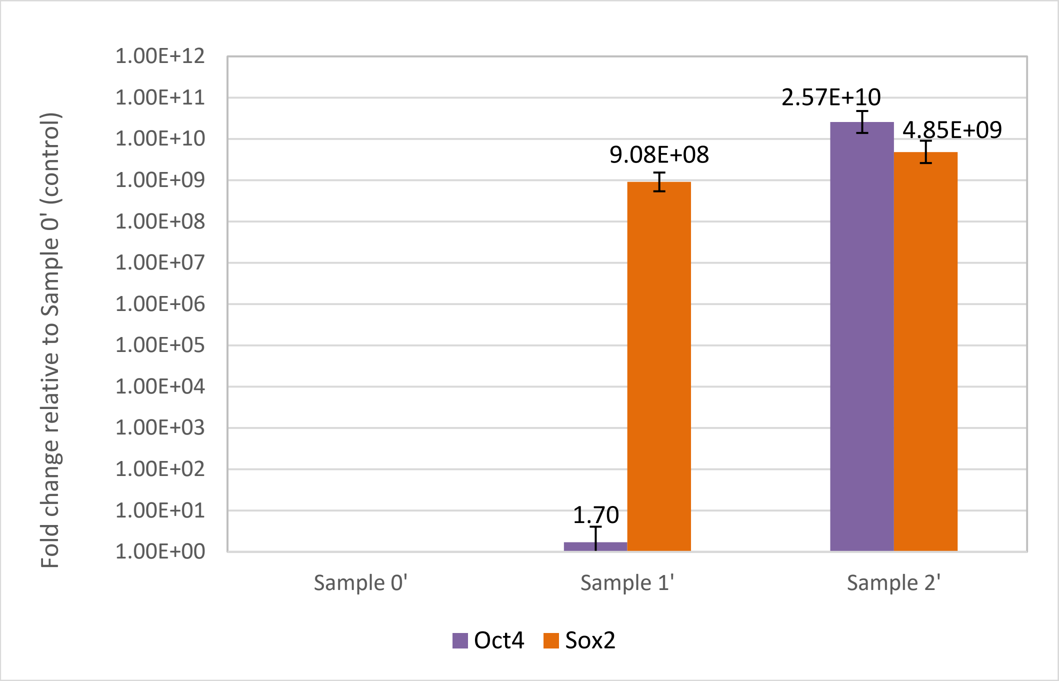

B

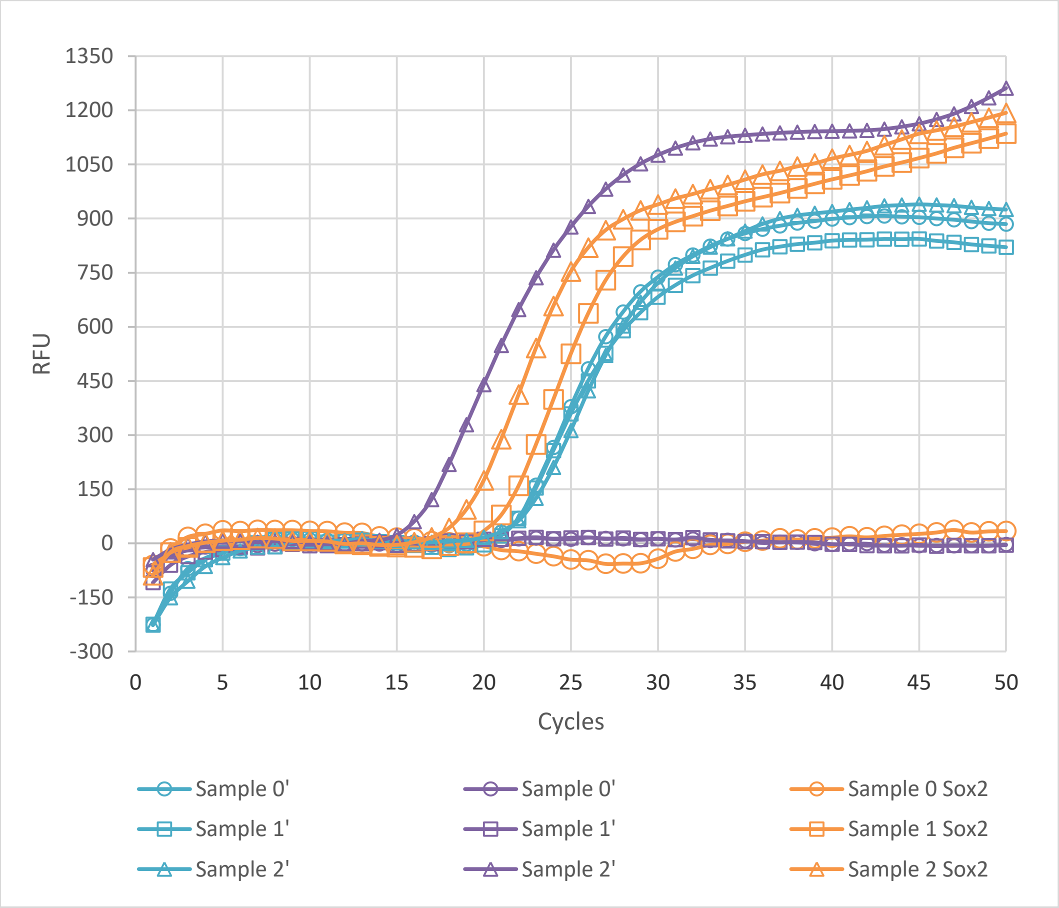

Figure S7. Bar chart diagram and overlay of amplification plots for samples 0′-2′. Sample 0′ (control without plasmids), Sample 1′ (BAP-GFP + BirA-SOX2), Sample 2 (BAP-OCT4 + BirA-SOX2): (A) Relative normalized expression of target genes; (B) real-time PCR amplification curves.
